# Elevated Lactate Dehydrogenase Levels Display a Poor Prognostic Factor for Non-Hodgkin’s Lymphoma in Intensive Care Unit: An Analysis of the MIMIC-III Database Combined With External Validation

**DOI:** 10.3389/fonc.2021.753712

**Published:** 2021-10-28

**Authors:** Jiaqian Qi, Chengyuan Gu, Weijuan Wang, Mengqi Xiang, Xiaochen Chen, Jianhong Fu

**Affiliations:** ^1^ National Clinical Research Center for Hematologic Diseases, Jiangsu Institute of Hematology, The First Affiliated Hospital of Soochow University, Suzhou, China; ^2^ Institute of Blood and Marrow Transplantation, Collaborative Innovation Center of Hematology, Soochow University, Suzhou, China; ^3^ Department of Hematology, Institute of Blood and Marrow Transplantation, Suzhou, China; ^4^ Key Laboratory of Thrombosis and Hemostasis of Ministry of Health, Suzhou, China; ^5^ Department of Medical Oncology, Sichuan Cancer Hospital, Medical School of University of Electronic Science and Technology of China, Chengdu, China; ^6^ State Key Laboratory of Radiation Medicine and Protection, Soochow University, Suzhou, China

**Keywords:** non-Hodgkin’s lymphoma (NHL), lactate dehydrogenase, MIMIC-III, prediction model, ICU - intensive care unit

## Abstract

**Background:**

Among the growing number of patients with hematologic neoplasms hospitalized in the intensive care unit (ICU), the largest proportion of these patients are diagnosed with lymphoma. However, less attention has been paid in the past to identifying critically ill patients and assessing the prognosis of patients in ICU. Traditional critical care-related scores have shown limitations and inaccuracy in predicting mortality risk.

**Methods:**

Patients diagnosed with diffuse large B-cell lymphoma (DLBCL) were searched for in the Marketplace for Information in Intensive Care Medicine III (MIMIC-III) database. We searched mortality within 28 days as the primary endpoint. Logistics regression was used to screen risk factors. A calibration curve was used for internal validation, and the ROC curve and AUC were used to compare the new model with traditional scores.

**Results:**

405 patients with DLBCL are enrolled in the project. Multivariate analysis shows the patients with the level of lactate dehydrogenase (LDH) > 327 U/L had an increased risk of 28-day mortality in ICU than others (OR = 13.04, p<0.01). Notably, length of ICU stay, LDH, creatinine, white blood cell counts, and APS III score are independent prognostic factors for patients with DLBCL in the ICU. Then, all these independent prognostic factors are selected into our prediction model. The new model has good accuracy (C-index=0.863) and a calibration curve, which improves clinical status concerning established ratings such as IPI, NCCN-IPI score, SOFA, APS III, and LODS. The results of a multicenter external validation including 124 DLBCL patients also showed that the new model was more accurate than all other models.

**Conclusions:**

The elevated level of LDH indicates a poor prognosis of patients with DLBCL in the ICU. Our risk score with crossed validation based on the level of LDH shows a significant prognostic value and may be a valuable tool for assessing the critically ill as well.

## Introduction

Diffuse large B-cell lymphoma is the most frequent type among invasive lymphoma, representing 30.0-40.0% of all non-Hodgkin’s lymphoma (NHL) (1, 2). It is regarded as a heterogeneous lymphoma in clinical features, tumor biology, and prognosis (3, 4).

Lactate dehydrogenase is a tetrameric enzyme that catalyzes the reversible transformation of pyruvic acid to lactate in the terminal stage of the glycolytic enzymatic pathway, together with nicotinamide adenine dinucleotide dehydrogenase (NADH) oxidation to NAD+. The LDH has emerged as a meaningful prognostic biomarker in neoplastic diseases. The elevated level of LDH has shown a strong negative correlation with survival in both HL and NHL patients. In a recent investigation, Garcia et al. (5, 6) found that the level of LDH >320 U/L, the clinical stage of lymphoma and age, had a critical prognostic impact on achieving complete remission (CR). This study demonstrated that elevated LDH was an independent prognostic factor for reducing disease-free survival and increasing the disease relapse rate. Ferraris et al. (7) also found that the level of LDH in serum was negatively related to survival in those patients with NHL in another study. Eventually, the elevated level of LDH has been listed as a risk factor in both the age-adjusted IPI and International Prognostic Index (IPI) for NHL (8). In addition, the elevated level of LDH is associated with disease progression and lower overall survival(OS) in multiple myeloma, independent of the international staging system (9). LDH has a great prognostic significance for patients with lymphoma, especially for stage III or IV, but it is not included in any ICU-related scores. ICU-related scores such as SOFA, APS III, LODS are widely used in patients with severe infections, but they may be less accurate and meaningful in the case of lymphoma in the ICU.

In this study, our purpose is to demonstrate the association of elevated LDH levels with higher mortality. The value of LDH is established by multivariate logistics analysis. Then, we obtain a model to determine the predictive value of patients with DLBCL.

## Methods

### Patients

We conducted a retrospective investigation based on an extensive database in the United States called the Marketplace for Medical Information in MIMIC-III (10). The MIMIC-III database comprises the comprehensive ICU patients hospitalized at Beth Israel Deaconess Medical Center between 2002 and 2012, which contains a total of 53,423 patients aged 16 years or older. In this database, a total of 561 patients were diagnosed with lymphoma, including 405 with DLBCL. One of the authors was granted access to this database and was in charge of the data extraction (accreditation number 10122491). This study complies with the Research Reports Using Observational Routine Health Data (RECORD) statement (11). We included patients in this study based on lymph node pathology and immunohistochemical findings showing a diagnosis of DLBCL. LDH was measured multiple times during each patient’s ICU stay and we have selected the highest value of LDH for inclusion in the data. From 2014, Feb 1^st^, to 2019 Jan 31^st^, 124 patients with DLBCL hospitalized in the ICU (**Figure S2**) and 81 non-ICU hospitalized patients (**Table S2**) from the First Affiliated Hospital of Soochow University and Sichuan Cancer Hospital were used for the multicenter external validation. The follow-up period was from the beginning of ICU admission to 1 year. Inclusion and exclusion criteria as well as treatment options are described in the **Supplementary Materials 1**.

### Statistical Analysis

The random forest algorithm and missing data were estimated using the rat package in RStudio (R version 4.0.2). Variables with normal distribution and skewness were expressed as mean ± standard deviation and median + IQR. Unpaired t-tests and Mann-Whitney U tests were used for comparisons between groups. The categorical variables were indicated as percentages and compared using the chi square test. Cumulative mortality was indicated by Kaplan-Meier curves and analyzed using the log-rank test. Multivariate and univariate survival analyses of overall survival (OS) were assessed using Cox regression models. The effect of covariates on prognosis was analyzed visually using forest plots. We included variables with p<0.015 in the prediction model and found that this as a criterion had the highest C-index and better accuracy.

To build accurate predictive models, the contribution of each covariate was quantified and presented as a Nomogram plot with 2000 internal validations. The consistency of the developed models was assessed using a calibration method. Statistical analyses were performed using the ‘mice’, ‘rms,’ ‘surminer’, and ‘ggplot2’ packages of RStudio (R version 4.0.2).

## Results

In total, 405 patients with DLBCL in ICU were selected for the investigation. The median age is 69 years (57-80 years), and 239 (59.0%) male patients were enrolled. The 28-day mortality rate and one-year mortality rate are 24.0% and 30.1%, respectively. The median duration of ICU stay is 2.97 days in all groups and 3.87 days in the 28-day death group (p=0.012). 150 (37%) patients were admitted to the ICU for respiratory failure, while 132 (33%) and 79 (20%) were for sepsis and cardiac insufficiency, respectively. The median LDH level is 327 U/L (interquartile range (IQR), 212-640) in all groups and 728 U/L (IQR,480-2133) in the 28-day death group. We also calculate the IPI, SOFA, ASC III, and LODS scores, and the median scores are 4 (IQR,2-7), 44 (IQR,32-58), and 4 (IQR,2-6), respectively (**Table 1**).

**Table 1:** Study Participant Characteristics at Enrollment.

Variables	Total (n = 405)	Cohort, median (IQR)		p-value
		alive (in 28 days) (n = 308)	dead (in 28 days) (n = 97)	
age	68.86 (57.42, 79.77)	68.72 (57.66, 78.59)	69.85 (56.6, 82.74)	0.239
gender, n (%)				0.516
F	166 (41)	123 (40)	43 (44)	
M	239 (59)	185 (60)	54 (56)	
BMI	26.74 (23.6, 30.6)	26.74 (23.57, 30.94)	25.81 (23.69, 30.24)	0.784
ICU staytime	2.97 (1.73, 6.09)	2.74 (1.66, 5.69)	3.87 (2.23, 8.66)	0.012
LDH	327 (212, 640)	283 (194, 399.75)	728 (480, 2133)	<0.001
lactate	1.5 (1.1, 2.1)	1.4 (1, 1.9)	1.8 (1.1, 3)	0.002
bicarbonate	22 (20, 25)	23 (20, 26)	21 (17, 23)	<0.001
bilirubin	0.6 (0.4, 1.2)	0.6 (0.4, 1.1)	0.9 (0.5, 2.1)	<0.001
creatinine	1 (0.8, 1.5)	1 (0.7, 1.4)	1.4 (0.9, 1.9)	<0.001
WBC	10.2 (6.1, 15.2)	9.65 (5.97, 13.9)	12.7 (6.7, 18)	0.003
hemoglobin	9.4 (8.2, 10.8)	9.5 (8.2, 10.9)	9.1 (8.3, 10.4)	0.172
PLT	148 (74, 245)	162.5 (82, 255.75)	99 (45, 202)	<0.001
PTT	32.9 (27.1, 42.8)	31.8 (26.73, 39.4)	40 (29.7, 50.4)	<0.001
INR	1.3 (1.1, 1.6)	1.3 (1.1, 1.5)	1.5 (1.2, 2.4)	<0.001
PT	14.5 (13.2, 17)	14.2 (13.07, 16.5)	16 (13.7, 21.5)	<0.001
SOFA	4 (2, 7)	4 (2, 6)	7 (4, 10)	<0.001
APSIII	44 (32, 58)	39 (30, 52)	58 (47, 81)	<0.001
LODS	4 (2, 6)	4 (2, 5)	6 (4, 9)	<0.001
Reasons for admission to ICU				0.046
respiratory failure	150 (37)	115 (37)	35 (36)	
sepsis	132 (33)	99 (32)	33 (34)	
cardiac insufficiency	79 (20)	54 (18)	25 (26)	
others	44 (11)	40 (13)	4 (4)	
Disease Stage				<0.001
I	216 (53)	182 (59)	34 (35)	
II	108 (27)	83 (27)	25 (26)	
III	63 (16)	37 (12)	26 (27)	
IV	18 (4)	6 (2)	12 (12)	
NCCN-IPI, n (%)				<0.001
0	56 (14)	50 (16)	6 (6)	
1	128 (32)	113 (37)	15 (15)	
2	86 (21)	76 (25)	10 (10)	
3	34 (8)	26 (8)	8 (8)	
4	24 (6)	11 (4)	13 (13)	
5	11 (3)	6 (2)	5 (5)	
6	51 (13)	23 (7)	28 (29)	
7	15 (4)	3 (1)	12 (12)	
IPI, n (%)				<0.001
0	75 (19)	65 (21)	10 (10)	
1	126 (31)	109 (35)	17 (18)	
2	76 (19)	64 (21)	12 (12)	
3	40 (10)	27 (9)	13 (13)	
4	18 (4)	13 (4)	5 (5)	
5	70 (17)	30 (10)	40 (41)	

IQR, interquartile range; WBC, white blood cell; Hb, hemoglobin; PLT, platelet; PTT, partial thromboplastin time; PT, prothrombin time; BMI, body mass index; ICU, intensive care unit; LDH, lactate dehydrogenase; INR, International Normalized Ratio.

By univariate analysis based on logistic regression, we realize that high LDH level may be a strong predictor of both 28-day and one-year mortality (Oddis Ratio [OR] 20.31, 95% confidence interval 10.06-46.91; P<0.001; OR 8.58, 95% CI 5.17-14.77). In addition, length of ICU stay, bilirubin, creatinine, hemoglobin(HB), white bicarbonate, blood cell (WBC), platelet (PLT), partial thromboplastin time(PTT), International Normalized Ratio(INR), prothrombin time (PT), SOFA, APS III, and LODS score are negatively correlated both with 28-day and one-year mortality (**Table 2**). All variants are statistically significant (P<0.01) after multivariate adjustment, and the increased level of the LDH group showed about an 18-fold increased risk of 28-day mortality (OR 18.33, 95% CI 8.55-44.58; P<0.001) (**Table 3**). Meanwhile, Cox regression also showed LDH level was an independent risk factor for patients’ prognosis in ICU (HR 8.62, 95% CI 4.12-18.00, P<0.001; adjusted by age and gender HR 8.86 95% CI 4.23-18.56, P<0.001) (**Table S1**). We then analyzed the effect of LDH on mortality within 28 days according to the different stages of the patients (I-II and III-IV) separately. We found that for patients with staging I or II, LDH > 285.5 U/L had a higher risk of death in the ICU (OR 12.64, 95% CI 5.65-33.79; P<0.001). In contrast, for patients with staging III or IV, LDH >997 U/L had a higher risk of death in the ICU (OR 2.67, 95% CI 1.10-6.74; P=0.033). However, analysis of data from patients not admitted to the ICU showed no significant effect of LDH on death within 28 days (OR 2.00, 95% CI 0.18-44.18; P=0.578).

**Table 2:** Univariate analysis for patients dead in 28 days or 1 year by logistic regression analysis.

Variables	dead in 28 days		dead in 1 year	
	OR (95% CI)	p-value	OR (95% CI)	p-value
age, >60 vs. ≤60 (year)	0.89 [0.55, 1.48]	0.656	0.82 [0.52, 1.31]	0.411
gender, female vs. male	1.20 [0.75, 1.90]	0.443	1.16 [0.75, 1.77]	0.51
BMI, >26.74 vs. ≤26.74	0.97 [0.61, 1.53]	0.885	0.99 [0.65, 1.52]	0.974
ICU staytime, >3 vs. <3 (day)	1.97 [1.24, 3.17]	0.004	2.63 [1.70, 4.11]	<0.001
LDH, >327 vs. ≤327 (U/L)	20.31 [10.06, 46.91]	<0.001	8.58 [5.17, 14.77]	<0.001
lactate, >1.5 vs. ≤1.5 (U/L)	1.79 [1.13, 2.85]	0.013	1.34 [0.88, 2.06]	0.173
bicarbonate >22 vs. ≤22 (U/L)	0.42 [0.26, 0.68]	<0.001	0.43 [0.27, 0.66]	<0.001
bilirubin, >0.6 vs. ≤0.6 (U/L)	2.57 [1.60, 4.19]	<0.001	2.09 [1.36, 3.24]	0.001
creatinine, >1 vs. ≤1 (U/L)	1.83 [1.16, 2.93]	0.011	1.68 [1.09, 2.58]	0.018
WBC, >10.2 vs. ≤10.2 (x10^9/L)	1.82 [1.14, 2.91]	0.012	1.72 [1.12, 2.65]	0.014
hemoglobin, <9.6 vs. ≥9.6 (U/L)	1.25 [0.79, 1.99]	0.348	1.44 [0.94, 2.22]	0.098
PLT <148 vs. ≥148 (x10^9/L)	2.45 [1.53, 3.98]	<0.001	2.46 [1.59, 3.84]	<0.001
PTT >32.7 vs. ≤32.7 (sec)	1.82 [1.15, 2.93]	0.012	1.70 [1.11, 2.63]	0.015
INR, >1.3 vs. ≤1.3	2.56 [1.60, 4.14]	<0.001	2.18 [1.42, 3.37]	<0.001
PT, >14.5 vs. ≤14.5 (sec)	2.44 [1.53, 3.95]	<0.001	2.36 [1.53, 3.67]	<0.001
Disease stage, IV or III vs. I or II	3.97 [2.36, 6.69]	<0.001	3.51 [2.12, 5.84]	<0.001
SOFA, >3.73 vs. ≤3.73	3.73 [2.31, 6.12]	<0.001	3.79 [2.43, 5.98]	<0.001
APSIII, >44 vs. ≤44	6.34 [3.73, 11.28]	<0.001	6.89 [4.22, 11.57]	<0.001
LODS, >4 vs. ≤4	4.02 [2.47, 6.69]	<0.001	4.14 [2.64, 6.59]	<0.001

OR, oddis ratio; WBC, white blood cell; Hb, hemoglobin; PLT, platelet; PTT, partial thromboplastin time; PT, prothrombin time; BMI, body mass index; ICU, intensive care unit; LDH, lactate dehydrogenase; INR, International Normalized Ratio.

**Table 3:** Multivariate analysis on risk factors for death in 28 days by logistic regression analysis.

Variables	OR (95% CI)	p-value
ICU staytime, >3 vs. <3 (day)	2.00 [1.07, 3.81]	0.031
LDH, >327 vs. ≤327 (U/L)	18.33 [8.55, 44.58]	<0.001
lactate, >1.5 vs. ≤1.5 (U/L)	1.28 [0.69, 2.38]	0.423
bicarbonate >22 vs. ≤22 (U/L)	0.80 [0.41, 1.54]	0.497
bilirubin, >0.6 vs. ≤0.6 (U/L)	1.54 [0.78, 3.04]	0.209
creatinine, >1 vs. ≤1 (U/L)	0.58 [0.28, 1.15]	0.122
WBC, >10.2 vs. ≤10.2 (x10^9/L)	2.51 [1.33, 4.84]	0.005
PLT <148 vs. ≥148 (x10^9/L)	1.02 [0.52, 2.00]	0.944
PTT >32.7 vs. ≤32.7 (sec)	1.25 [0.63, 2.48]	0.514
INR, >1.3 vs. ≤1.3	2.23 [0.59, 9.33]	0.252
PT, >14.5 vs. ≤14.5 (sec)	0.56 [0.14, 2.08]	0.402
SOFA, >3.73 vs. ≤3.73	1.01 [0.45, 2.25]	0.976
APSIII, >44 vs. ≤44	3.62 [1.60, 8.45]	0.002
LODS, >4 vs. ≤4	1.71 [0.78, 3.74]	0.176

OR, oddis ratio; WBC, white blood cell; Hb, hemoglobin; PLT, platelet; PTT, partial thromboplastin time; PT, prothrombin time; BMI, body mass index; ICU, intensive care unit; LDH, lactate dehydrogenase; INR, International Normalized Ratio.

**Figure 1:**
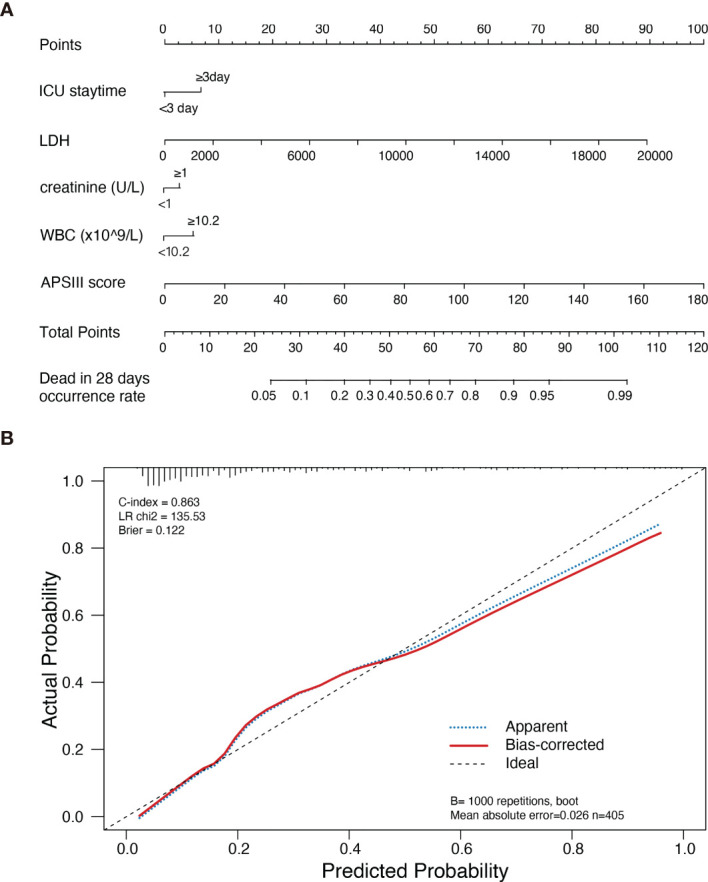
Nomogram of new model estimates in patients with DLBCL in ICU and its predictive performance. **(A)** Nomogram of death risk estimates in DLBCL patients with different variants in ICU. When using the Nomogram plot, locate the position of each variable on the axis, then draw a line on the integral axis, sum the scores of all variables, draw the line at the lower line of the Nomogram plot, and draw a line on the total integral axis to determine the death probability. **(B)** Validity of the Nomogram in estimating the predictive performance of risk in patients with DLBCL (n=405).

**Figure 2:**
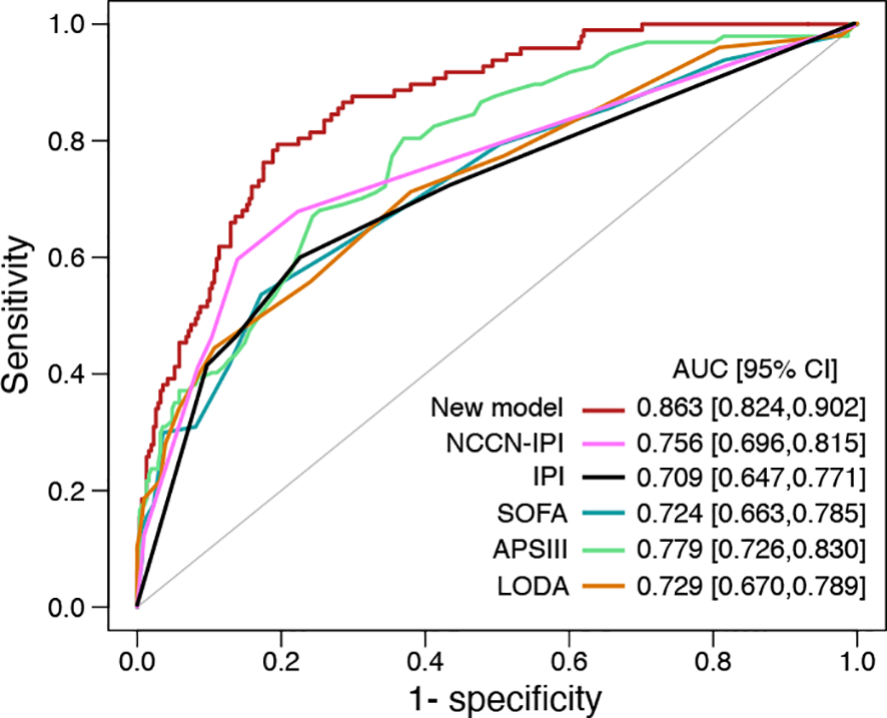
ROC curve for the new model, IPI, NCCN-IPI, SOFA, ASP III, and LODS score.

**Figure 3:**
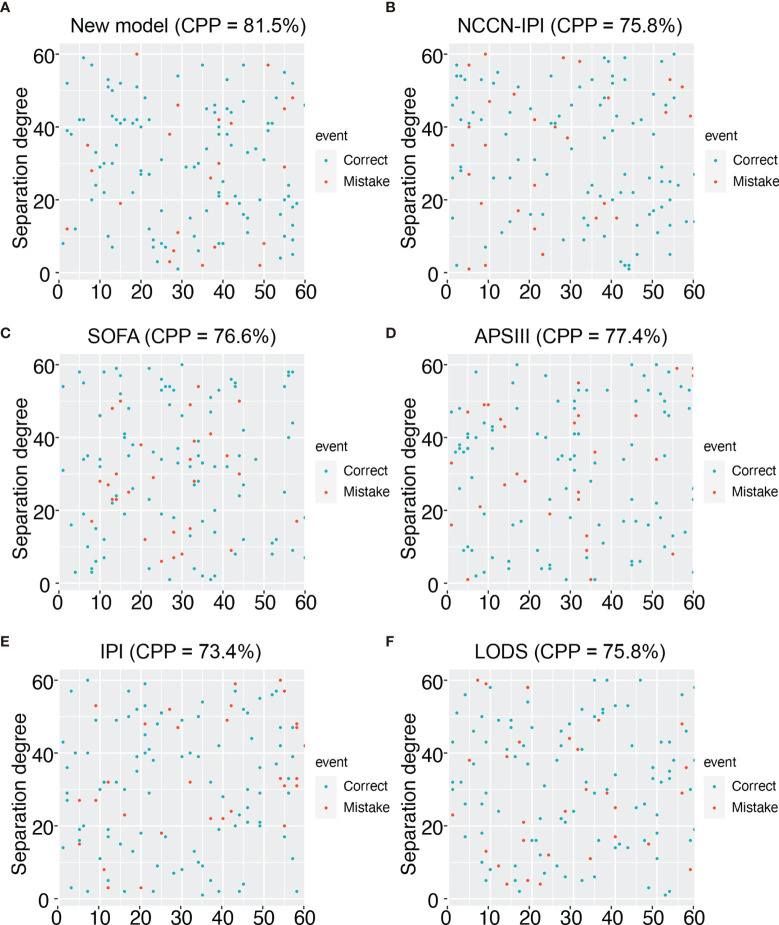
Prediction accuracy of external data for different models. **(A)** Prediction accuracy chart of the new model. **(B)** Prediction accuracy chart of the NCCN-IPI score. **(C)** Prediction accuracy chart of the SOFA score. **(D)** Prediction accuracy chart of the ASP III score. **(E)** Prediction accuracy chart of the IPI score. **(F)** Prediction accuracy chart of LODS score.

28-day mortality is widely used in ICU treatment as an appropriate and meaningful endpoint. Predicting the 28-day mortality is important and meaningful for clinicians to adjust the therapeutic regimen to improve patient outcomes. Univariate analysis shows the length of ICU stay, bicarbonate, bilirubin, creatinine, WBC, HB, PLT, PTT, INR, PT, SOFA, APS III, and LODS scores are risk factors for 28-day mortality. We included variables that were significant (p-value <0.05) in the univariate analysis in the multifactorial analysis. The length of ICU stay, LDH, creatinine, WBC, and APS III score were independent variables of 28-day mortality in DLBCL (**Table 3**). To calculate the weight of each factors to the 28-day mortality, a nomogram is generated in **Figure 1A** and the R2 of this model is 0.462. This model shows a good correction (C-index=0.863) (**Figure 1B**). Notably, ROC curves for patients with DLBCL in ICU also show that our new model is much more accurate of (AUC 0.863, 95% CI 0.824-0.902) compared to the NCCN-IPI(AUC=0.756, 95% CI 0.696-0.815), IPI score(AUC=0.709, 95% CI 0.647-0.771), ASP III (AUC=0.779, 95% CI 0.726-0.830), SOFA (AUC=0.724, 95% CI 0.663-0.875), and LODS models (AUC=0.729, 95% CI 0.670-0.789) (**Figure 2**). We constructed a simple linear model based on these significant variables through Fisher simple linear discriminant function and calculated the AUC (0.681[0.641,0.720]). We found that the new model was more accurate than the simple linear model (**Figure S1**). In addition, we collected multicenter clinical data for external validation. We found that the new model has higher predictive accuracy (81.5%) than the NCCN-IPI (75.8%), IPI (73.4%), SOFA (76.6%), APS III (77.4%), and LODS (75.8%) score (**Figures 3A–F**).

## Discussion

Our results show that the high level of LDH (>327 U/L) is a highly relevant predictor of increased mortality within 28 days in patients with DLBCL. When creating the new prediction model for the patients with DLBCL in ICU, the parameter, especially the level of LDH, is included, and the duration of ICU stays, WBC, creatinine, and ASP III score. We tested the accuracy with externally validated data and found that the prediction accuracy of the new model is above 80%. So, the validity and accuracy of this model were reasonable. Furthermore, the new model has a larger area under the AUC curve than traditional ICU scores such as SOFA, APS III, and LODS. For patients admitted to the ICU, death within 28 days is a very important clinical endpoint to judge the effectiveness of a patient’s treatment in the ICU. That is why we have adopted this clinical endpoint for lymphoma patients treated in the ICU. Our analysis also revealed that for non-ICU hospitalised lymphoma patients, the majority of patients do not die within 28 days. Therefore, the analysis of 28-day mortality in this group of patients is not meaningful or statistically significant.

LDH is an adverse prognostic biomarker for several hematological diseases and solid tumors. In a recent meta-analysis, Petrelli et al. found that elevated LDH levels may predict poor prognosis in various solid tumors; this effect was most pronounced in prostate, lung, renal cell, nasopharyngeal, and gastric cancers (12). Also, elevated LDH levels were the most independent risk factor in predicting decreased survival in the grading score of melanoma, regardless of the number and size of metastases. The degree of elevated LDH levels was negatively correlated with OS in melanoma patients (13). In a retrospective investigation of prognostic contribution in patients with breast cancer, increased serum LDH level is associated with 2-fold increased mortality. In contrast, serum LDH levels above 2-fold ULN were associated with 6-fold high mortality (14). Notably, elevated LDH level has an essential prognostic significance for male germ cell tumor patients. The LDH, α-fetoprotein (AFP), and ß human chorionic gonadotropin (ß-hCG) are included in the Germ Cell Carcinoma Collaborative Group’s risk stratification system as serum tumor markers with prognostic significance (15). LDH is an active enzyme in the pathway of anaerobic metabolism. It is a member of the oxidoreductase class, enzyme commission number EC 1.1.1.27. The enzyme’s function is to facilitate the reversible conversion of lactate to pyruvate with simultaneous reduction of NAD+ to NADH and vice versa (16). Thus it promotes the proliferation and progression of lymphoma. CD20 monoclonal antibody enables patients to achieve remission and long-term survival (17, 18). Therefore, in addition to lymphoma, LDH may be a prognostic predictor for patients with many types of tumors in the ICU.

The tumor microenvironment is widely implicated in tumorigenesis and interacts with surrounding cells through the network of extracellular matrix, soluble factors, and cells (19–21). Lactic acid is one of the key soluble factors in the microenvironment. Excess lactate produced by the lactate dehydrogenase-A (LDH-A) in tumor cells is exported from the cytoplasm by the monocarboxylate transporter (MCT), which plays a vital role in facilitating proton-linked lactate transportation across membranes. The extracellular pH drops to 6.0-6.5 due to circulating acidosis of the tumor microenvironment caused by the exported lactic acid (20). Lactate has various immunosuppressive effects. In the tumor microenvironment, immunosuppressive cells and immunostimulatory cells are present. Cancer-associated immunosuppressive cells, including tumor-associated macrophages (TAMs) and Bone marrow-derived suppressor cells (MDSCs), are considered cancer-associated immunosuppressive cells. MDSCs are composed of macrophages, monocytes, granulocytes, and dendritic cells, a population of immature bone marrow cells that suppress and regulate T cells. TAMs are a population of pro-inflammatory cells that promote tumor progression through cytokines and chemokines like IL-23, IL-6, IL-12, and TNF-α (22). Therefore, for the modulation of LDH levels, the clearance of tumor cells by the immune system may be improved. In the intensive care unit, LDH is influenced by multiple stressors, and its predictive value compared to IPI and NCCN-IPI is advantageous, which are simple predictors of tumor prognosis. Our findings corroborate this. Since we searched the critical care database, some information such as a relapsed refractory lymphoma was unavailable. This was a retrospective clinical study, and its level of evidence was not strong enough. Moreover, since the data were obtained from a database, their previous chemotherapy regimen and the time of application were not available, which is one of the limitations of this study. Also, the number of externally validated cases is relatively small. More patients need to be included in the future to validate this new model.

## Conclusions

In conclusion, our results show that the elevated level of LDH predicted high 28-day mortality in patients with DLBCL treated in ICU. A cross-validated multivariate score shows a good agreement in predicting 28-day mortality in patients with DLBCL in ICU.

## Data Availability Statement

The raw data supporting the conclusions of this article will be made available by the authors, without undue reservation.

## Ethics Statement

Informed consent was obtained from all patients or their immediate family members. All research programs are in line with the guidelines of the Ethics Committee of Soochow University and follow the Declaration of Helsinki.

## Author Contributions

JQ designed and performed research studies, analyzed the data, and wrote the manuscript. CG and WW completed research studies and analyzed data. MX contributed to the data analysis and manuscript writing. JF and XC contributed to the research design, data analysis, manuscript writing, and study supervision. All authors contributed to the article and approved the submitted version.

## Funding

This work was supported by the National Natural Science Foundation of China (Nos. 81873432 and 81670132), grants from the Jiangsu Province of China (Nos. ZDRCA2016047), Jiangsu Provincial Special Program of Social Development (No. SBE2016740635), and the Priority Academic Program Development of Jiangsu Higher Education Institutions.

## Conflict of Interest

The authors declare that the research was conducted in the absence of any commercial or financial relationships that could be construed as a potential conflict of interest.

## Publisher’s Note

All claims expressed in this article are solely those of the authors and do not necessarily represent those of their affiliated organizations, or those of the publisher, the editors and the reviewers. Any product that may be evaluated in this article, or claim that may be made by its manufacturer, is not guaranteed or endorsed by the publisher.

## Abbreviations

IQR, interquartile range; OR, oddis ratio; WBC, white blood cell; Hb, hemoglobin; PLT, platelet; PTT, partial thromboplastin time; PT, prothrombin time; BMI, body mass index; ICU, intensive care unit; LDH, lactate dehydrogenase; INR, International Normalized Ratio.
