# Supplementary material for: Elevated Lactate Dehydrogenase Levels Display a Poor Prognostic Factor for Non-Hodgkin’s Lymphoma in Intensive Care Unit: An Analysis of the MIMIC-III Database Combined With External Validation
Source: Front Oncol. 2021 Oct 28;11:753712. doi: 10.3389/fonc.2021.753712 (PMC8581292; doi:10.3389/fonc.2021.753712)
Supplement: Supplementary file 1 [file DataSheet_1.docx]

**Criteria**

**Inclusion Criteria:**

1. Histologically confirmed DLBCL with extrinsic involvement
2. Age ≥ 18 years
3. Measurable disease of at least 15mm(node)/10mm（extranodal）
4. ECOG performance status 0-2
5. Subjects of childbearing or child-fathering potential must be willing to practice birth control from the time of enrollment on this study until the follow-up period of the study

**Exclusion Criteria:**

1. Accepted major surgery within 4 weeks before treatment;
2. Diagnosis of primary mediastinal lymphoma or primary CNS lymphoma;
3. Previous history of indolent lymphoma;
4. Prior malignancy (other than DLBCL), except for cured malignant tumors with no active lesions for 3 years; Adequate treatment of inactive lesions in non-melanoma skin cancer, malignant tonsilloma or carcinoma in situ;
5. Evidence of complications or medical conditions, including but not limited, that may interfere the conduct of the study or place the patient at serious risk: myocardial infarction within 6 months of screening、uncontrolled or symptomatic arrhythmias) and/or significant lung disease;
6. HIV infection and/or active hepatitis B or active hepatitis C;
7. Pregnant or breasting-feeding women;

**Treatment**

R-CHOP: Rituximab + Cyclophosphamide + Doxorubicine + Vincristine + Prednisone

Drug: Rituximab

cycles of 21 days - 375mg/m²

Other Name: Mabthera

Drug: Cyclophosphamide

cycles of 21 days - 750 mg/m²

Drug: Doxorubicin

cycles of 21 days - 50mg/m²

Drug: Vincristine

cycles of 21 days - 1.4mg/m²

Drug: Prednisone

cycles of 21 days - 40mg/m²
