# Supplementary figures and images for: Elevated Lactate Dehydrogenase Levels Display a Poor Prognostic Factor for Non-Hodgkin’s Lymphoma in Intensive Care Unit: An Analysis of the MIMIC-III Database Combined With External Validation
Source: Front Oncol. 2021 Oct 28;11:753712. doi: 10.3389/fonc.2021.753712 (PMC8581292; doi:10.3389/fonc.2021.753712)

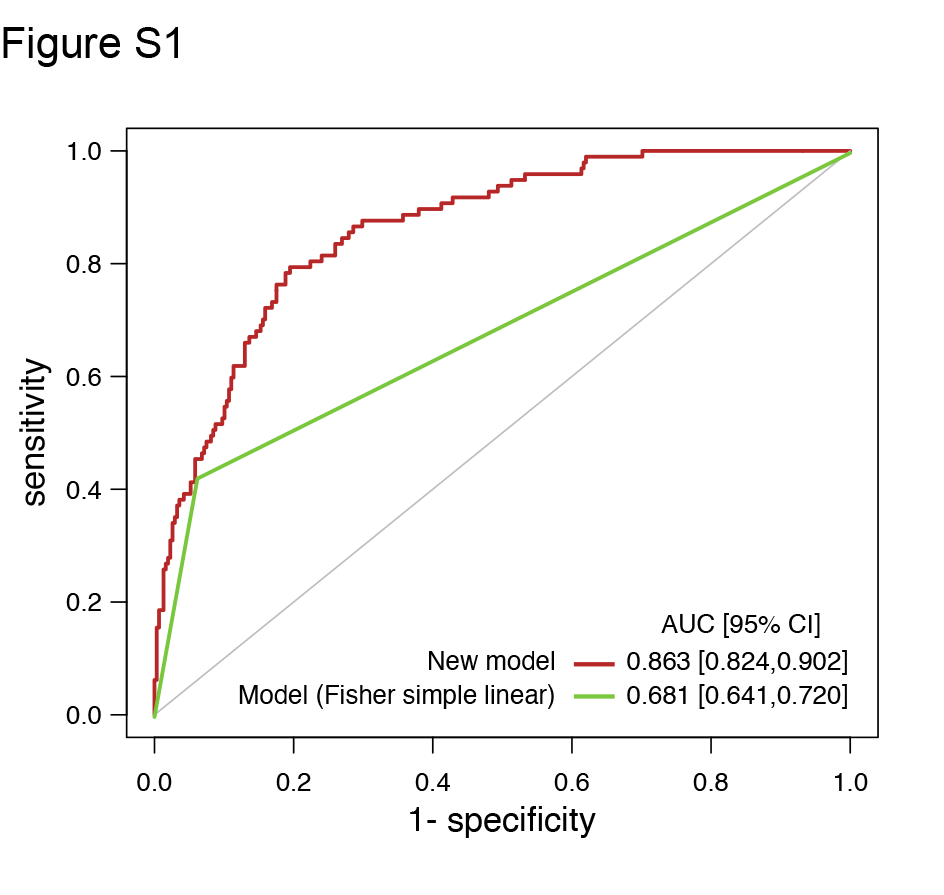

Supplement: Supplementary file 2 [file Image_1.tif]

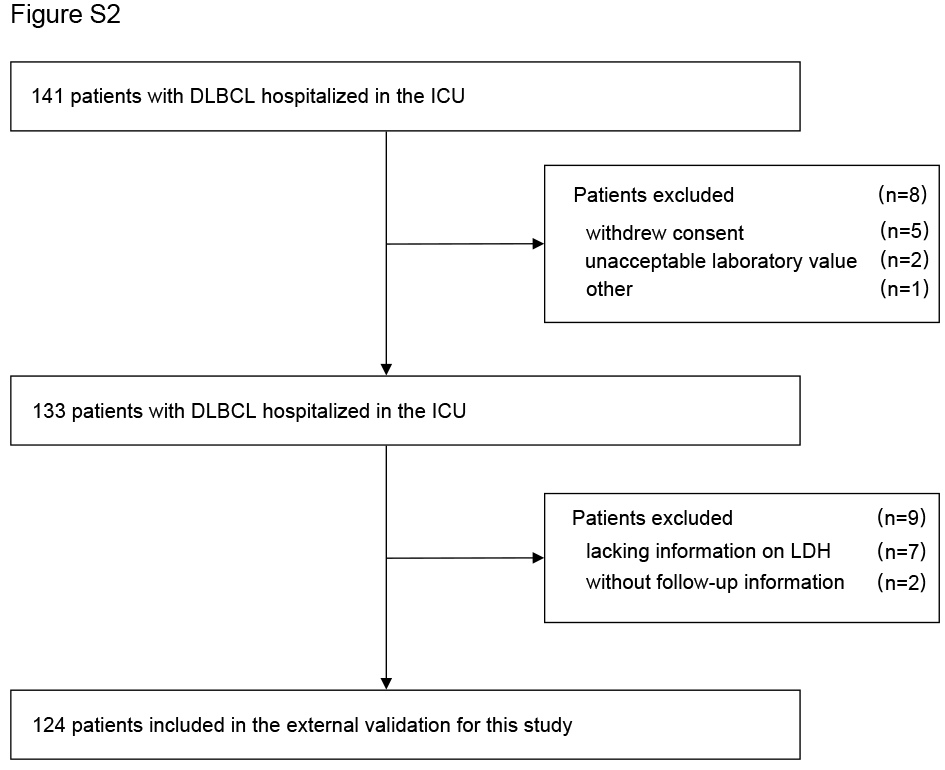

Supplement: Supplementary file 3 [file Image_2.tif]
